# Supplementary material for: Mechanisms governing the pioneering and redistribution capabilities of the non-classical pioneer PU.1
Source: Nat Commun. 2020 Jan 21;11:402. doi: 10.1038/s41467-019-13960-2 (PMC6972792; doi:10.1038/s41467-019-13960-2)
Supplement: Supplementary file 7 — Source data [file 41467_2019_13960_MOESM7_ESM.zip › Source_Data/FigureS3/FigureS3D_MotifsHEPG2/knownResults.html]

/misc/data/analysis/project\_PU1/predictionHepG2/motifs/PU1\_Kmeans15.all.redhomer - Homer Known Motif Enrichment Results


# Homer Known Motif Enrichment Results (/misc/data/analysis/project\_PU1/predictionHepG2/motifs/PU1\_Kmeans15.all.redhomer)

Homer *de novo* Motif Results  
Gene Ontology Enrichment Results  
Known Motif Enrichment Results (txt file)  
Total Target Sequences = 40737, Total Background Sequences = 39195

|  |  |  |  |  |  |  |  |  |  |  |  |
| --- | --- | --- | --- | --- | --- | --- | --- | --- | --- | --- | --- |
| Rank | Motif | Name | P-value | log P-pvalue | q-value (Benjamini) | # Target Sequences with Motif | % of Targets Sequences with Motif | # Background Sequences with Motif | % of Background Sequences with Motif | Motif File | SVG |
| 1 | A G T C C A T G T G A C G T A C G T A C G A T C A G T C G A C T C T G A A T C G G C A T A C T G C T A G | 1-YGCCCYCTRGTGG,BestGuess:BORIS(Zf)/K562-CTCFL-ChIP-Seq(GSE32465)/Homer(0.943) | 1e-141 | -3.254e+02 | 0.0000 | 1825.0 | 4.48% | 584.0 | 1.49% | motif file (matrix) | svg |
| 2 | T C G A G A C T A C T G G C T A A T G C C G A T G T A C C T G A A G C T G A T C G A T C | 2-ATGASTCAYHN,BestGuess:Fra1(bZIP)/BT549-Fra1-ChIP-Seq(GSE46166)/Homer(0.995) | 1e-102 | -2.351e+02 | 0.0000 | 2175.0 | 5.34% | 953.2 | 2.43% | motif file (matrix) | svg |
| 3 | G C A T C T A G A G C T C A G T A C G T C T G A G T A C G C T A A G T C C G T A C T A G | 3-TGTTTACWYAG,BestGuess:FOXA1/MA0148.3/Jaspar(0.971) | 1e-77 | -1.785e+02 | 0.0000 | 3837.0 | 9.42% | 2319.8 | 5.92% | motif file (matrix) | svg |
| 4 | C G A T C T A G T C A G G T C A T A G C G A C T A G C T C A G T T C A G T C G A G T C A G T A C | 4-TGRACTTTGAAC,BestGuess:HNF4G/MA0484.1/Jaspar(0.958) | 1e-73 | -1.685e+02 | 0.0000 | 2692.0 | 6.61% | 1480.4 | 3.78% | motif file (matrix) | svg |
| 5 | T C A G A T G C T G A C T A C G T A C G C T G A C G T A T C G A | 11-GCCGGAAA,BestGuess:Elk4(ETS)/Hela-Elk4-ChIP-Seq(GSE31477)/Homer(0.943) | 1e-50 | -1.163e+02 | 0.0000 | 2058.0 | 5.05% | 1167.7 | 2.98% | motif file (matrix) | svg |
| 6 | C A G T T C G A G A C T A C G T C T A G G A T C C T A G G A T C T G C A T G C A | 7-NRTTGCGCAA,BestGuess:CEBPE/MA0837.1/Jaspar(0.958) | 1e-40 | -9.318e+01 | 0.0000 | 2483.0 | 6.10% | 1578.7 | 4.03% | motif file (matrix) | svg |
| 7 | C A T G T C A G A C G T A C T G C G T A C A G T A C T G G T A C C G T A C G T A A G C T T G A C | 14-KRTGATGCAATM,BestGuess:Atf4(bZIP)/MEF-Atf4-ChIP-Seq(GSE35681)/Homer(0.949) | 1e-39 | -9.208e+01 | 0.0000 | 1008.0 | 2.48% | 477.7 | 1.22% | motif file (matrix) | svg |
| 8 | C T A G C T A G A T G C G A T C G T A C A G T C C A T G A G T C A G T C A G T C G T A C | 7-GGCCCCGCCCC,BestGuess:POL003.1\_GC-box/Jaspar(0.962) | 1e-29 | -6.805e+01 | 0.0000 | 2059.0 | 5.06% | 1348.0 | 3.44% | motif file (matrix) | svg |
| 9 | C T A G A C G T G C A T C T G A G T C A G C A T A T G C C G T A C A G T G A C T C G T A C T G A G A T C G A C T | 6-GTTAATBATTAACY,BestGuess:HNF1A/MA0046.2/Jaspar(0.965) | 1e-27 | -6.407e+01 | 0.0000 | 501.0 | 1.23% | 201.1 | 0.51% | motif file (matrix) | svg |
| 10 | A G T C G A C T A G C T C G A T A C T G G C T A C G A T T A G C G C A T A T G C G C A T A C T G | 10-CTTTGATCTSTB,BestGuess:TCF7L2/MA0523.1/Jaspar(0.916) | 1e-22 | -5.115e+01 | 0.0000 | 3457.0 | 8.49% | 2611.0 | 6.66% | motif file (matrix) | svg |
| 11 | C T A G T G A C T A C G T G A C G T C A C A G T A C T G A G T C A C T G G A T C C T G A | 19-GCGCATGCGCA,BestGuess:NRF(NRF)/Promoter/Homer(0.982) | 1e-16 | -3.860e+01 | 0.0000 | 264.0 | 0.65% | 99.9 | 0.25% | motif file (matrix) | svg |
| 12 | A T G C A C G T A C G T C G T A A C G T A G T C C G A T | 15-CTTATCT,BestGuess:Gata6(Zf)/HUG1N-GATA6-ChIP-Seq(GSE51936)/Homer(0.974) | 1e-16 | -3.859e+01 | 0.0000 | 3746.0 | 9.20% | 2959.5 | 7.55% | motif file (matrix) | svg |
| 13 | C T A G T C A G A G C T C A T G C T G A A G T C T C A G G A C T G T A C C G T A | 15-GRTKACGTCA,BestGuess:Atf1(bZIP)/K562-ATF1-ChIP-Seq(GSE31477)/Homer(0.926) | 1e-11 | -2.712e+01 | 0.0000 | 1254.0 | 3.08% | 895.2 | 2.28% | motif file (matrix) | svg |
| 14 | G A C T C T A G A C T G C A T G T C G A C T G A G C A T G A C T A C T G A G C T C T G A A C T G A C G T A G C T | 16-TGGGARHTGTAGTY,BestGuess:GFY(?)/Promoter/Homer(0.932) | 1e-10 | -2.510e+01 | 0.0000 | 215.0 | 0.53% | 93.0 | 0.24% | motif file (matrix) | svg |
| 15 | C A T G A C T G A G C T T G A C T G C A C T G A A C T G A C T G G A C T G A T C C G T A A T G C | 12-GGTCAAGGTCAC,BestGuess:Nr5a2(NR)/Pancreas-LRH1-ChIP-Seq(GSE34295)/Homer(0.973) | 1e-9 | -2.262e+01 | 0.0000 | 1467.0 | 3.60% | 1104.6 | 2.82% | motif file (matrix) | svg |
| 16 | C T G A C T A G A G T C A G T C C G T A G C T A A C G T T A G C C T G A T C A G T C G A | 16-ARCCAATCRGV,BestGuess:NFY(CCAAT)/Promoter/Homer(0.953) | 1e-9 | -2.101e+01 | 0.0000 | 895.0 | 2.20% | 633.0 | 1.61% | motif file (matrix) | svg |
| 17 | A G C T A C G T G A T C G A T C T A C G C T A G C T A G G T C A C G T A C G A T | 18-TTCCGGGAAT,BestGuess:Stat3(Stat)/mES-Stat3-ChIP-Seq(GSE11431)/Homer(0.942) | 1e-4 | -9.784e+00 | 0.0001 | 2723.0 | 6.69% | 2360.3 | 6.02% | motif file (matrix) | svg |
| 18 | C A T G C T G A G T A C C G T A A C G T A C G T A G T C A G T C C G T A | 13-GACATTCCA,BestGuess:TEAD3/MA0808.1/Jaspar(0.958) | 1e-2 | -6.670e+00 | 0.0013 | 3143.0 | 7.72% | 2806.9 | 7.16% | motif file (matrix) | svg |
